# Health care expenditure and health outcome nexus: new evidence from the SAARC-ASEAN region

**DOI:** 10.1186/s12992-018-0430-1

**Published:** 2018-11-22

**Authors:** Mohammad Mafizur Rahman, Rasheda Khanam, Maisha Rahman

**Affiliations:** 1 Faculty of Business, Education, Law and Arts, University of Southern Queensland, Toowoomba, Australia; 2 Faculty of Medicine, University of Queensland, Brisbane, Australia

**Keywords:** Healthcare expenditure, Health status outcomes, Panel data, SAARC, ASEAN, I10, I15, I18, C23

## Abstract

**Background:**

The total health expenditure (as a percentage of GDP) and health outcomes in the region of South Asian Association for Regional Cooperation (SAARC) and Association for South East Asian Nations (ASEAN) are lower than that of the OECD region and the world. This study investigated the relationship between different types of healthcare expenditures (public, private and total) and three main health status outcomes - life expectancy at birth, crude death rate and infant mortality rate - in the region.

**Methodology:**

Using the World Bank data set for 15 countries over a 20-year period (1995–2014), a panel data analysis was conducted where relevant fixed and random effect models were estimated to determine the effects of healthcare expenditure on health outcomes. The main variables studied were total health expenditure, public health expenditure, private health expenditure, GDP per capita, improved sanitation, life expectancy at birth, crude death rate and infant mortality rate.

**Results:**

Total health expenditure, public health expenditure and private health expenditure significantly reduced infant mortality rates, and, the extent of effect of private health expenditure was greater than that of public health expenditure. Private health expenditure also had a significant role in reducing the crude death rate. Per capita income growth and improved sanitation facilities also had significant positive roles in improving population health in the region.

**Conclusions:**

Health expenditure in the SAARC-ASEAN region should be increased as our results indicated that it improved the health status of the population in the region. Public sector health funds must be appropriately and efficiently used, and accountability and transparency regarding spending of public health funds should be ensured. Finally, government and private institutes should implement appropriate strategies to improve sanitation facilities.

## Introduction

Enriched human capital is considered an important factor for achieving desired economic growth and development in any country [1, 2]. According to the neoclassical growth model, growth in human capital, in terms of education and health, positively affects per capita income in the long run [2]. Bloom and Canning [3, 4] and Bloom et al. [5] identified four mechanisms through which healthier individuals contribute to the economy: (i) at the workplace, healthier individuals are more productive and thus generally earn a higher income, (ii) they are able to retire later and take less sick leave due to overall good health and so they are able to work longer, (iii) they are more likely to invest in their own education and training which then enhances their productivity; and (iv) they are likely to save and invest more with the expectation of a longer life. Therefore, health is an integral part of sustainable development, and attempts for its improvement should always be the main development goal of a nation [6]. Furthermore, good health ensures economic security for the individuals themselves and their families [7], and provides a sense of empowerment that adds value to human life [8].

It is vital for all countries to appropriately invest in their health sector. Evidence shows that investing in health significantly benefits the economy [5, 9]. For example, a WHO report [10] revealed that for every increase in life expectancy at birth by 10%, the economic growth rate increased by 0.35% per year. Similarly, ill health is considered as a huge financial burden, and it is the major cause of 50% of the growth differential between developed and developing countries. Despite the importance of health investment, health expenditure in developing regions like South Asian Association for Regional Cooperation (SAARC) and Association for South East Asian Nations (ASEAN) is still under-represented in government budgets due to scarcity of resources [11].

Realizing the importance of population health and its contribution to the national economy, researchers have been conducting studies to explore the link between healthcare expenditure and health sector outcomes for more than two decades. However, most of these studies are based on developed countries (see, for example, Anderson and Poullier [12], Babazono and Hillman [13], Beger and Messer [14], Cochrane et al. [15], Crémieux et al. [16], Crémieux et al. [17], Elola et al. [18], Hitiris and Possnett [19], Or [20], Nixon and Uimann [21], Wolfe and Gabay [22]); but such studies on developing countries/regions are limited. Furthermore, to the best of our knowledge, no such study that uses macro data, has yet been conducted in the SAARC-ASEAN region. In addition, the health sectors and health expenditure patterns have changed significantly during the last decade. Therefore, a recent study that uses macro data and is specific to the SAARC-ASEAN region is warranted. This was our main rationale for conducting current research on a panel of 15 countries that will contribute to this gap in the literature. Furthermore, the debate on the relationship between health care expenditure and health outcomes is still inconclusive (Novignon et al. [1]). This study will help mitigate this debate by providing new evidence from a new region. Additional contributions of this study are that: the endogeneity issue of the concerned variables has been addressed, and the effects of healthcare expenditures on three health status indicators have been investigated as well, whilst most other studies investigated the effects of only one or two health status indicators. Hence, our study has a broader coverage.

**Fig. 1:**
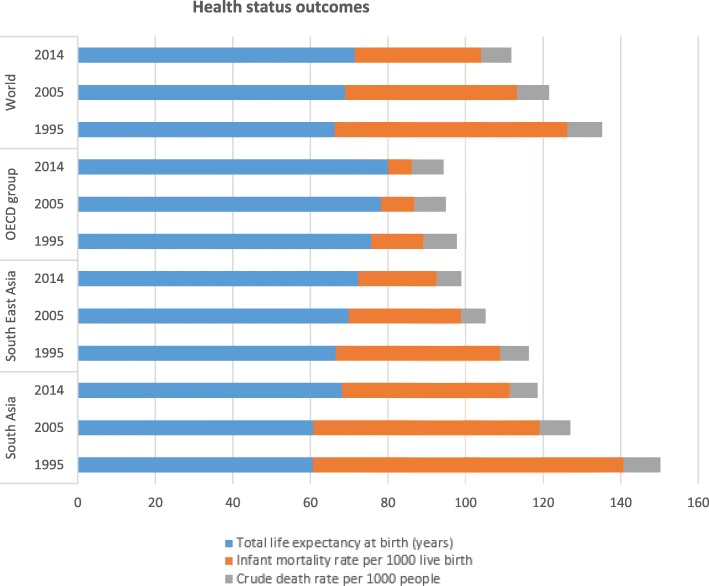
Health status outcomes for selected regions (selected years). Source: Word Development Indicators, World Bank, 2016

The aims of this study were: (i) to explore the impact of total health care expenditure on three health outcomes - life expectancy at birth, crude death rate and infant mortality rate in the SAARC-ASEAN region, and (ii) to investigate the differentiated influences of public versus private health care expenditure on these health outcomes. We have also explored the impact of two controlled variables: real GDP per capita and improved sanitation facilities. The rationale for selecting the three health outcomes (life expectancy at birth, crude death rate, infant mortality rate) was that the current status of these health outcomes is relatively poor in the countries of the SAARC-ASEAN region, compared to that of developed countries and the world in general (see Fig. 1).

The sections below in this paper are structured as follows: section 2 reviews the past empirical literature; section 3 briefly highlights the regional profiles; section 4 describes the methodology, data and model; section 5 presents empirical results; section 6 discusses the results; and, section 7 concludes the paper with policy implications.

### Literature review

Past empirical studies on the relationship between healthcare expenditure and health sector outcomes provide conflicting views. For example, Rana, et al. [23], Anand and Ravallion [24], Patricio et al. [25] and Imoughele and Ismaila [26] revealed a positive relationship between public healthcare expenditure and health sector performance for 30 OECD countries, Sri Lanka, Russia and Nigeria, respectively. On the other hand, Filmer and Pritchett [27], Musgrove [28] and Kim and Moody [29] found no relationship between these variables. Filmer and Pritchett [27] identified that rather than public healthcare expenditure, the level of poverty, income inequality, female education and other socio-economic factors were the main determining factors of child mortality. Furthermore, a World Bank study on Indian states using panel data for the period 1980–1999 found no effects of healthcare expenditure on infant mortality rate [30], which is similar to the findings of Burnside and Dollar [31]. Some other studies like Zakir and Wunnava [32], Nolte and Mckec [33] and Young [34] also found no significant and consistent relationship between health spending and health outcomes.

In a separate study in Brazil, Alves and Belluzzo [35] employed static panel data models to explore the determinants of infant mortality rate using census data for the period of 1970–2000. They found that education level, sanitation and poverty were significantly related to infant mortality rates. The same outcomes were also observed by the studies of Meara [36], Currie and Moretti [37] and Filmer [38].

In relation to the positive effects of healthcare expenditure on health outcomes, more empirical evidence has been found worldwide. In a study on 47 African countries between 1999 and 2004, Anyanwu and Erhijakpor [9] found that total healthcare expenditure significantly affected health outcomes: for every 1% increase of total health care expenditure per capita, there was a 2.1 and 2.2% decrease in under-five and infant mortality rates, respectively. Similarly, Akinkugbe and Mohanoe [39] also found a significant effect of healthcare expenditure, along with other variables, on health outcomes. Cross-country data analysis by Gupta et al. [40] on the relationship between public health expenditure and health status showed significant and stronger effects for the poorer population. In another study on 50 developing and transition countries, Gupta et al. [41] revealed that health expenditure reduced child mortality rates in 1994. Similarly, Issa and Ouattara [42] found a strong inverse relationship between health spending and infant mortality rate in a panel study of 160 countries, where they also segregated health expenditure into public and private spending. The same results on infant and child mortality rates were also observed by Paxson and Schady [43] and Wang [44], in relation to private health expenditure and public health expenditure, respectively. Significant inverse relationships between health care expenditure and mortality rates were also revealed by: Berger and Messer [14] in 20 OECD countries over the period of 1960–1992, Gani [45] in Pacific Island countries over the period of 1990–2002, Farag [46] in the Eastern Mediterranean region during the period of 1995–2006, and Novignon, and Lawanson [47] in sub-Saharan Africa over the period of 1995–2011.

Furthermore, Barenberg et al. [48], Maruthappu et al. [49] and Kumar et, al. [50] found similar results of the inverse relationship between public health spending and infant/child mortality rate for India, 176 countries and India, respectively. Maruthappu et al. [49] noted that low income countries, compared with higher income countries, experienced higher infant/child mortality rates due to reduction in public health spending. Kumer et al. [50] found a marginal effect of − 0.13 on under-five mortality and − 0.08 on infant mortality in relation to public health spending.

Recently, Arthur and Oaikhenan [51] conducted a study on 40 sub-Saharan African (SSA) countries using World Bank data, where they found an inelastic but significant effect of health expenditure on health outcomes. Their findings indicated that health expenditure reduced mortality rates and improved life expectancy marginally. They found that mortality rate was related to public health expenditure, whereas life expectancy was linked with private health expenditure. The study of Ashiabi et al. [52] on 40 SSA countries also found similar results, implying that public health expenditure improved infant and under-five mortality rates. The findings of Farahani et al. [53] in India also showed that a 10% increase in public health spending reduced the death rate by about 2% across all age groups. In another study on East African countries, Bein et al. [54] found a strong positive effect of total health expenditure on total life expectancy, and an inverse association between healthcare expenditure and mortality rate, implying the improvement of health outcomes with increased spending. The positive relationship between health expenditure and life expectancy was also revealed by the study of Jaba et al. [55] for all groups of countries regardless of their income level; Ranabhat et al. [56] also observed a positive effect of universal health coverage, where health expenditure is a major component, on the life expectancy in 193 countries. In contrast, Heuvel and Olaroiu [57] did not find health expenditure to be a main determinant of life expectancy in 31 European countries.

The above literature review indicates that there is a lack of studies, specific to the SAARC-ASEAN region, on the relationship between health care expenditure and health outcomes, particularly a study that uses macro data. Moreover, instead of examining one health outcome indicator, the present study has examined three health outcome indicators separately. Therefore, this study has a much broader scope, and it is unique in using macro data in the SAARC-ASEAN region, which will certainly contribute to the existing gap in the literature and have important policy implications.

### Brief profile of the SAARC-ASEAN region

The economic, political and social structures of the countries in the SAARC-ASEAN region are more or less similar, and therefore, comparable. SAARC or South Asia has a population of 1, 744 million, which is 23.75% of the world population. The ASEAN or South-East Asian population is 625 million, which is 8.8% of the world population. Together the SAARC-ASEAN region comprises 32.55% of the world’s population, and 5.81% of the world’s GDP [58]. Hence, the region is a significant player in the world stage, and therefore, any research that seeks to improve the economy and population health of that region is critical. The present study addresses this need.

Table 1 highlights the trend of health care expenditure of South Asia (SAARC countries) and South East Asia (ASEAN countries) against the OECD group and the world for selected years. It is observed that although total health expenditure (as percentage of GDP) has an increasing trend over the years in the SAARC-ASEAN regions, it is still far below the OECD region’s and the world’s health expenditure trends. In 2014, while the share of total health expenditure was 12.36% in OECD countries and 9.97% in the world, this was only 4.37% in South Asia and 4.72% in South East Asia. Between 2005 and 2014, the growth rate of total health expenditure in OECD countries was 12.5%, but it was only 7% in South Asia and 11% in South East Asia.

**Table 1:** Trend in health care expenditures for selected regions (selected years)

Regions	Total health expenditure (% of GDP)			Public health expenditure (% of government expenditure)			Out of pocket health expenditure (% of private expenditure on health)		
	1995	2005	2014	1995	2005	2014	1995	2005	2014
South Asia	3.76	4.07	4.37	4.43	4.80	5.25	92.17	89.36	89.41
South East Asia	3.69	4.24	4.72	7.64	8.18	10.42	86.41	84.60	80.07
OECD group	9.23	10.99	12.36	13.41	16.23	17.76	41.42	37.58	36.01
World	8.52	9.80	9.97	–	15.39^a^	15.61^b^	45.90	43.33	45.80

Note: ^a^ data for 2010

^b^ data for 2011

Source: World Development Indicators, World Bank, 2016

The condition of public health expenditure (as a percentage of government expenditure) is very poor in South Asia. It is less than one third of that of the OECD countries, and around half of that of South East Asian countries. It is also only one third of the world average public health expenditure. Although South East Asia has a better health expenditure profile than South Asia, it is still half of that of the OECD and world average (see Table 1). The growth rate of public health expenditure over the years is unconvincing for South Asia (a very small increase from 4.80% in 2005 to 5.25% in 2014) against the increase of South East Asia (an increase from 8.18% in 2005 to 10.42% in 2014), and the OECD countries (an increase from 16.23% in 2005 to 17.76% in 2014). It is plausible that poverty in the region, especially in South Asia, may be the main reason for low public health expenditure. This raises major concern for expected heath status outcomes in the region.

Out of pocket health expenditure (as a percentage of private expenditure on health) in both South Asia and South East Asia has decreased slightly over the years, along the lines of OECD countries. However, this out of pocket spending is still very high in SAARC and ASEAN regions compared to the OECD and world average. Out of pocket health expenditure in South Asia and South East Asia is approximately double that of the world average. While this share of out of pocket health expenditure was only 36.01% in the OECD group in 2014, it was 89.41% for South Asia and 80.07% for South East Asia. The study of Rancic and Jakovljevic [59] also found very high proportion of private expenditure on health, driven mainly by out-of-pocket spending, for Bangladesh and the Philippines among the Next − 11 emerging countries. This high proportion of out-of-pocket health spending is a major concern, as it will aggravate the existing poverty and compromise the welfare of the vast population of the SAARC-ASEAN region.

Although the study of Jakovljevic and Getzen [60] noted the increasing trend of greater investment in health care particularly in India, Bangladesh, Pakistan, Indonesia, the Philippines and Vietnam, it is still far below than some other comparable countries. For example, total health expenditure (% of GDP) of India was the lowest (3.97%) among the BRICS countries in 2013. The per capita government and private expenditures on health were also the lowest in India among the BRICS countries which were just $69 and $146, respectively [61]. Dieleman et al. [62] forecasted that, in low-income countries, per capita health spending would remain low at $154 in 2030 and $195 in 2040.

Figure 1 shows the trend of the health status outcomes of the SAARC and ASEAN regions in comparison with the OECD group and the world. It is observed that although total life expectancy at birth (years) has an increasing trend over the years in the SAARC-ASEAN region, it is always lower than that of the OECD group. In particular, total life expectancy in South Asia is always lower than that of the world average, highlighting the poor health status in the region. Whilst the world average total life expectancy is 71.45 years in 2014, it is only 68.12 years in South Asia.

Infant mortality rate per 1000 live births has improved tremendously in the SAARC-ASEAN region over the years. It has decreased from 80.10 per 1000 live births in 1995 to 43.30 per 1000 live births in 2014 in South Asia. In South East Asia, it has decreased to 20.26 per 1000 live births in 2014 from 42.44 per 1000 live births in 1995. However, it is still much higher than the OECD average (6.09 per 1000 live births) and the world average (32.60 per 1000 live births) in 2014, especially for South Asia, implying again the poor health status in the study region.

The crude death rate per 1000 people in the region has a similar trend to the OECD group and the world. Over the years, the crude death rate has been declining slightly. In 2014, it was 7.16 per 1000 people for South Asia, 6.27 per 1000 people for South East Asia, 8.10 per 1000 people for the OECD group and 7.75 per 1000 people for the world. These crude death rates may be misrepresentations of actual crude death rates in South Asia vs. South-East Asia vs. OECD countries vs. the world. For example, OECD countries, in general, have a larger ageing population and the population of SAARC-ASEAN regions mostly consist of young people. Hence comparatively, the crude death rate may be over-represented in OECD countries due to a larger proportion of the population being elderly.

## Methodology

In the literature, different estimation methods (e.g. cross sectional analysis, panel, autoregressive distributed lag model, etc.) have been used to analyse the relationship between healthcare expenditure and health status outcomes. This paper used a panel data estimation method. For studies that cover multiple countries, panel data estimation is the best approach to follow. Furthermore, panel data analysis has the following advantages over cross sectional analysis and time series analysis: i) panel data provides a more accurate inference of model parameters via more degrees of freedom and more sample variability; hence econometric estimates are improved, efficient and reliable [63], ii) panel data controls the impact of omitted variables, iii) panel data takes into account the inter-individual differences, and, iv) different data periods can be used for different countries with unbalanced panel data. Therefore, our chosen method of a panel data analysis for this research is justified.

Following Novignon et al. [1], we adopted a health outcome model as follow:

1 $$ {y}_{it}={H}_{it}\beta +{\varepsilon}_t,t=1\dots ..T $$

2 $$ {\upvarepsilon}_{\mathrm{t}}=\upmu \mathrm{Z}+\mathrm{v} $$

where *y*_*it*_ is a vector of dependent variables in country i at time t, H is a vector of exogenous variables, including the constant, and β is a vector of coefficients. *ε*_*t*_ is a vector of random error terms. Baltagi et al. [64] propose two components of the error process such as time variant and reminder error process. The error term is spatial weights matrix, Z, and contains spatial autocorrelation parameter, μ.

We have also considered the effects of two controlled variables: real per capita income and improved sanitation facilities. These two variables are chosen based on earlier literature and the availability of data in the countries of study. Economic theory suggests that people with a higher level of income have increased spending capacity (as purchasing power increases) on better quality and healthy food, as well as on better health services. It is also expected that good sanitation facilities can provide better health outcomes. Therefore, to investigate the effects of health care expenditure, real per capita income and sanitation on health outcomes, we specify the following equation:

3 $$ {\mathrm{HS}}_{\mathrm{it}}\kern0.5em =\upalpha +{\upbeta}_1{\mathrm{THE}}_{\mathrm{it}}+{\upbeta}_2{\mathrm{GDP}}_{\mathrm{it}}+{\upbeta}_3{\mathrm{SAN}}_{\mathrm{it}}+{\upvarepsilon}_{\mathrm{it}} $$

where, HS denotes the three health outcome variables, namely, total life expectancy at birth (years), infant mortality rate (per 1000 live births) and crude death rate (per 1000 people). THE is total health expenditure (as % of GDP), and GDP represents gross domestic product per capita (constant 2005 US$) and SAN is improved sanitation facilities.

Total health expenditure is the summation of two types of health expenditure: public and private health expenditures. Public health expenditure includes recurrent and capital spending from government budgets and social or compulsory insurance funds. On the other hand, private health expenditure includes private health insurance premiums, direct payments or out-of-pocket health expenditure [1, 65]. Both private and public health expenditures have different effects on health status. For example, an increase in out-of-pocket health expenditure, which is a type of private health expenditure, reduces the individual’s spending ability on other goods and services (including other health goods and services). This may lead to more poverty and thus the cycle continues with increased poor health status due to lack of money to spend on additional health goods and services. On the other hand, an increase in public health expenditure may worsen government budget deficit, but it will decrease the burden of individual private health expenditure. Increased public health expenditure contributes to improved societal health, which allows for improved human capital that eventually leads to higher economic growth [66]. Thus we analysed the individual impact of these two components (public and private health expenditures) on health outcomes using the following equation:

4 $$ {\mathrm{HS}}_{\mathrm{it}}\kern0.5em =\upalpha +{\upbeta}_1{\mathrm{PUB}}_{\mathrm{it}}+{\upbeta}_2{\mathrm{PRI}}_{\mathrm{it}}+{\upbeta}_3{\mathrm{GDP}}_{\mathrm{it}}+{\upbeta}_4{\mathrm{SAN}}_{\mathrm{it}}+\kern0.5em {\upvarepsilon}_{\mathrm{it}} $$

where, PUB and PRI represent public health expenditure and private health expenditure, respectively. All variables are in natural logarithmic form. The subscripts i and t represent country and time, respectively.

Firstly, we ran a fixed effect model generalised least squares (GLS) and a random effect model GLS (with cross-section weights). Baltagi et al. [64] argue that the random effect model is more suitable when the error term is considered not serially correlated with the reminder error and there is no spatial serial dependence of error terms. Cameron and Trivedi [67] argue that fixed effect may be used to control endogeneity in panel data where endogeneity arises owing to time-invariant omitted variables. We conducted the Hausman Test to investigate whether the fixed effect or random effects model was the most appropriate model. In addition, we addressed the potential endogeneity issue by adopting the Panel Generalized Method of Moments (GMM). Arellano and Bond [68] propose that the use of instrumental variable GMM mitigates the endogeneity problem with explanatory variables. Furthermore, GMM is very useful in estimating extensions of the basic unobserved effects [69].

Initially, we collected annual data from 1960 to 2014 (55 years) from the World Development Indicators (WDI), which is a World Bank database for 17 countries of the South Asia (SAARC) and South East Asia (ASEAN) regions. These countries were Bangladesh, Bhutan, Brunei Darussalam, Cambodia, India, Indonesia, Lao PDR, Malaysia, Maldives, Myanmar, Nepal, Pakistan, Philippines, Singapore, Sri Lanka, Thailand and Vietnam. The chosen variables for collected data were total life expectancy at birth (years), infant mortality rate (per 1000 live births), crude death rate (per 1000 people), per capita real GDP, improved sanitation facilities (% of population with access), total health expenditure (% of GDP), public health expenditure (% of government expenditure) and private health expenditure i.e. out-of-pocket health expenditure (% of private expenditure on health). Due to lack of data for all countries and for all years, we had to limit the study to 15 countries excluding Myanmar and Brunei Darussalam for the period of 1995–2014 (20 years).

## Results

Table 2 provides the mean and standard deviation of life expectancy at birth (LIF), mortality rate (MOR), death rate (DEA), per capita real GDP (GDP), total health expenditure (THE), public health expenditure (PUP), private health expenditure (PRI) and improved sanitation facilities (SAN).

**Table 2:** Descriptive statistics of variables

	LIF	DEA	MOR	GDP	THE	PRI	PUB	SAN
Bangladesh								
Mean	67.37	6.43	53.57	509.00	2.73	94.83	7.87	50.06
Std. Dev.	2.98	0.89	15.29	121.80	0.30	2.25	0.96	6.13
Bhutan								
Mean	63.85	7.79	48.37	1343.95	5.59	98.07	12.26	37.89
Std. Dev.	4.26	1.55	14.93	420.86	1.30	2.25	3.26	8.86
Cambodia								
Mean	62.06	8.14	58.36	468.01	5.83	94.30	9.73	24.13
Std. Dev.	4.44	1.75	22.47	161.03	0.83	8.62	3.82	10.27
India								
Mean	64.33	8.25	57.46	768.06	4.29	89.99	4.39	30.10
Std. Dev.	2.33	0.68	11.99	244.75	0.18	1.69	0.30	5.85
Indonesia								
Mean	67.06	7.24	35.30	1336.29	2.50	74.82	5.00	51.38
Std. Dev.	1.17	0.06	8.47	259.47	0.39	1.56	0.75	6.03
Lao PDR								
Mean	61.32	8.77	72.63	504.03	3.63	78.71	6.11	42.55
Std. Dev.	3.18	1.53	14.30	158.28	1.03	10.62	2.15	16.96
Malaysia								
Mean	73.43	4.58	7.95	5587.07	3.51	76.59	5.60	92.96
Std. Dev.	0.85	0.17	1.69	929.23	0.45	1.98	0.58	2.40
Maldives								
Mean	72.74	4.31	25.03	4223.96	7.86	65.26	15.49	86.86
Std. Dev.	3.78	0.88	15.09	703.87	2.05	15.06	4.04	9.74
Nepal								
Mean	64.76	7.80	49.76	325.83	5.85	84.81	11.19	28.93
Std. Dev.	3.47	1.25	14.50	52.21	0.44	7.35	2.52	9.66
Pakistan								
Mean	63.82	8.34	81.43	693.06	2.83	87.07	4.13	44.90
Std. Dev.	1.44	0.67	8.96	78.54	0.27	5.65	0.51	10.59
Philippines								
Mean	67.18	6.28	27.77	1230.63	3.76	81.75	8.12	66.80
Std. Dev.	0.67	0.20	3.17	206.24	0.58	2.78	0.88	3.94
Singapore								
Mean	79.54	4.48	2.73	29,060.14	3.49	94.70	9.04	99.84
Std. Dev.	2.03	0.24	0.67	5641.49	0.69	1.17	2.13	0.21
Sri Lanka								
Mean	72.73	6.69	12.39	1319.45	3.66	84.04	7.30	85.87
Std. Dev.	2.02	0.46	2.88	374.54	0.29	4.59	1.62	6.14
Thailand								
Mean	72.10	7.09	16.24	2883.15	4.93	68.76	16.72	92.18
Std. Dev.	1.53	0.42	4.05	534.58	0.89	9.77	4.85	1.45
Vietnam								
Mean	73.94	5.65	23.44	705.23	5.60	89.97	8.48	60.49
Std. Dev.	1.15	0.11	4.01	209.81	0.80	6.26	2.47	9.92

Notes: *LIF* = Life expectancy at birth, *DEA* = Crude death rate, *MOR* = infant mortality rate; *GDP* = Gross domestic product (per capita), *THE* = Total health expenditure, *PUB* = Public health expenditure, *PRI* = Private health expenditure, *SAN* = Sanitation facilities

### The effects of health care expenditures on life expectancy

The effect of health care expenditure on life expectancy was investigated using the fixed and random effect models, and the results are reported in Table 3.

**Table 3:** Effects of health care expenditures on life expectancy at birth (LIF)

Model	Fixed Effect		Random Effect	
C	3.3306 (142.61)^a^	3.3777 (59.17)^a^	3.3807 (144.15)^a^	3.4243 (60.30)^a^
LNTHE	0.0011 (0.23)		0.0055 (1.23)	
LNPUB		−0.0015 (− 0.45)		0.0001 (0.04)
LNPRI		−0.0093 (− 0.88)		− 0.0088 (− 0.85)
LNGDP	0.0740 (13.67)^a^	0.0750 (14.21)^a^	0.0608 (12.56)^a^	0.0631 (13.19)^a^
LNSAN	0.0911 (16.32)^a^	0.0890 (15.72)^a^	0.1004 (19.03)^a^	0.0972 (17.97)^a^
R-squared	0.9805	0.9805	0.8888	0.8891
F-Statistic	885.05	885.51	772.96	579.11
Observations	294	294	294	294
Cross-section included	15	15	15	15

Notes: ^a, **,^ and

^*^denote significance level at 1, 5 and 10%, respectively. Figures in the parentheses are t-statistics

### The effects of health care expenditures on death rate

The results regarding the effects of health care expenditures on death rate are noted in Table 4 below.

**Table 4:** Effects of health care expenditures on death rate (DEA)

Model	Fixed Effect		Random Effect	
C	3.4010 (35.49)^a^	3.8843 (16.78)^a^	3.4273 (35.77)^a^	3.9320 (17.01)^a^
LNTHE	0.0910 (4.82)^a^		0.0861 (4.66)^a^	
LNPUB		0.0645 (4.69)^a^		0.0632 (4.65)^a^
LNPRI		−0.1065 (−2.51)^b^		−0.1100 (−2.60)^a^
LNGDP	0.0016 (0.07)	0.0111 (0.52)	−0.0063 (− 0.32)	0.0017 (0.09)
LNSAN	−0.4173 (−18.20)^a^	− 0.4385 (−19.11)^a^	−0.4098 (−18.96)^a^	−0.4307 (− 19.56)^a^
R-squared	0.9637	0.9647	0.7866	0.7946
F-Statistics	430.76	417.07	356.22	279.50
Observations	294	294	294	294
Cross-section included	15	15	15	15

Notes: ^a, b,^ and ^*^ denote significance level at 1, 5 and 10%, respectively. Figures in the parentheses are t-statistics

### The effect of health care expenditures on infant mortality rate

The estimated results of health care expenditures on infant mortality rate are presented in Table 5 below.

**Table 5:** Effects of health care expenditures on infant mortality rate (MOR)

Model	Fixed Effect		Random Effect	
C	10.1249 (52.39)^a^	11.3396 (22.58)^a^	10.0440 (47.78)^a^	11.2206 (22.15)^a^
LNTHE	−0.2683 (−7.05)^a^		−0.2749 (−7.32)^a^	
LNPUB		−0.0833 (−2.79)^a^		−0.0888 (−3.00)^a^
LNPRI		−0.2383 (−2.59)^a^		−0.2405 (−2.62)^a^
LNGDP	−0.7840 (−17.51)^a^	−0.8579 (− 18.48)^a^	−0.7581 (− 18.12)^a^	−0.8209 (− 18.89)^a^
LNSAN	− 0.2124 (−4.59)^a^	−0.1709 (− 3.43)^a^	−0.2332 (−5.23)^a^	−0.1996 (−4.14)^a^
R-squared	0.9893	0.9879	0.8467	0.8267
F-Statistics	1495.82	1247.25	533.76	344.70
Observations	294	294	294	294
Cross-section included	15	15	15	15

Notes: ^a, **,^ and ^*^ denote significance level at 1, 5 and 10%, respectively. Figures in the parentheses are t-statistics

### Addressing the potential endogeneity issue

We have estimated the panel GMM results to address the potential endogeneity problem. The obtained results are noted in Table 6 below. We found similar results of the fixed and random effect models reported in Table 3–5 above.

**Table 6:** The results of panel Generalized Method of Moments (GMM)

Dependent variable	LIF		DEA		MOR	
C	3.3306 (149.24)^a^	3.3777 (84.62)^a^	3.4273 (35.88)^a^	3.8843 (19.85)^a^	10.1249 (68.28)^a^	11.3396 (25.50)^a^
LNTHE	0.0011 (0.27)		0.0861 (4.32)^a^		−0.2683 (−6.69)^a^	
LNPUB		−0.0015 (− 0.43)		0.0645 (3.86)^a^		−0.0833 (−2.88)^a^
LNPRI		−0.0093 (−1.45)		−0.1065 (−3.66)^a^		− 0.2383 (− 2.85)^a^
LNGDP	0.0740 (13.54)^a^	0.0750 (14.41)^***^	−0.0063 (− 0.31)	0.0111 (0.51)	− 0.7840 (− 19.65)^a^	−0.8579 (−19.35)^a^
LNSAN	0.0911 (23.36)^a^	0.0890 22.05	−0.4098 (−27.80)^a^	− 0.4385 (− 27.61)^a^	−0.2124 (−4.86)^a^	−0.1709 (− 4.11)^a^
R-squared	0.9804	0.9805	0.7866	0.9646	0.9893	0.9879
Observations	294	294	294	294	294	294
Cross-section included	15	15	15	15	15	15

Notes: ^a, **,^ and ^*^ denote significance level at 1, 5 and 10%, respectively. Figures in the parentheses are t-statistics

## Discussion

Table 2 shows that the highest (79.54) and the lowest (61.32) mean of LIF were reported in Singapore and Lao PDR, respectively. The highest and lowest mean death rates were 8.77 and 4.33 in Lao PDR and Maldives, respectively. The lowest mean value of mortality (2.73) and the highest mean values of GDP and SAN (29,060.14 and 99.84, respectively) were reported in Singapore. Whilst the lowest mean of total health expenditure was reported in Indonesia (2.50), it was the highest in Maldives (7.86). The highest and the lowest means of public health expenditure were in Thailand (16.72) and India (4.39), respectively. The highest mean private health expenditure was in Bhutan (98.07), and the lowest mean was in the Maldives (65.26). In Cambodia, the availability of sanitation facilities was minimal among the sample countries.

Table 3 results reveal that an increase of total health expenditures had no impact on life expectancy at birth (LIF), consistent with the findings of Filmer and Pritchett [27] and Barlow and Vissandjee [70], but contradicting the findings of Novignon et al. [1]. In addition, public health care expenditure (PUB) and private health care expenditure (PRI) also had no impact on life expectancy at birth. This may be explained by the fact that life expectancy is affected by other factors, such as diet, lifestyle and environment, which are not directly related to the health care system [21]. Per capita income (GDP) and sanitation (SAN) were found to improve life expectancy at birth. The corrected random effect Hausman specification test confirmed that fixed effect estimate was more appropriate (Chi-Sq. statistic is 31.87) in this estimate. The fixed effect model was significant with an R-square of 98%, and F-statistic of 885.

Table 4 reveals that total health expenditure (THE) had a positive impact on death rate; an increase in THE was more likely to increase death rate with a 1% level of significance. When we split the THE into public and private, public health expenditure (PUB) was found to increase death rate; whereas, private health care expenditure (PRI) reduced death rate. Whilst public health care expenditure increased the death rate by about 0.06 in both the fixed and random effects models, private health care expenditure reduced death rate by 0.11 per 1000 people in the fixed and random effect models at 1% significance level, partially consistent with the findings of Novignon et al. [1] from sub-Saharan Africa. However, the studies of Berger and Messer [14] and Hitiris and Possnett [19] on OECD countries revealed that health expenditure reduced mortality rate in developed countries. This discrepancy between our findings and other studies may be due to the fact that there is limited good governance for utilising public health expenditure in SAARC-ASEAN countries. If resources in the public sector are inefficiently used, and corruption prevails, the likely outcomes on health status will not be achieved [71]. Furthermore, public health care expenditure compared to private health care expenditure in developing countries are manifold; since the sign of public health expenditure is positive, the effect of total health expenditure is also found positive (see Akinci et al. [72], for example). Additionally, while per capita GDP had no effect on death rate (DEA), improved sanitation facilities reduced the death rate. The corrected random effects of Hausman specification test confirmed that the random effect estimate was more appropriate (Chi-Sq. statistic is 5.45) in this case. The random effect model was significant with an R-square of 79%, and F-statistic of 356.

Table 5 shows that total health care expenditure was more likely to reduce the infant mortality rate and that a 1% increase in total health care expenditure (as percentageof GDP) led to a reduction in infant mortality rate by around 0.27 per 1000 live births in the fixed and random effect models at a 1% significance level.. When the total healthcare expenditure is divided into public and private, both expenditures decreased the infant mortality rate, as expected. Both real per capita GDP and sanitation facilities reduced the infant mortality rate as well. These results are consistent with the findings of Kumar et al. [50], Barenberg et al. [48], Crémieux et al. [17], Elola and Vicente [18], Novignon, et al. [1], Issa and Ouattara [42], Paxson and Schady [43] and many others. The extent of effect on infant mortality rate was higher with private health expenditure (0.24) than public health expenditure (0.09). The corrected random effect Hausman specification test confirmed that the random effect estimate was more appropriate (Chi-Sq. statistic is 3.07) in this case. The random effect model was significant with an R-square of 85%, and F-statistic of 534.

Overall, our main results indicated that total health care expenditure significantly reduced the number of infant mortalities per 1000 live births. The separate effects of public and private health expenditures on infant mortality rate were also negative in the sample of selected countries. While health care expenditure had no effect on life expectancy at birth, private health expenditure had an inverse relationship with death rate in the selected sample countries. Here, public and private health care expenditures showed conflicting effects on the death rate.

## Conclusion and policy implications

This study has explored the role of healthcare expenditure on three important health status outcomes: life expectancy at birth, crude death rate and infant mortality rate, in the SAARC-ASEAN region. A panel data set of 15 countries for 20 years (1995–2014) was used. The separate effects of private and public healthcare expenditures on health outcomes were also examined. Furthermore, two control variables, GDP per capita and improved sanitation facilities, were also added in the selected models as explanatory variables.

Our findings reveal that total health expenditure had a significant effect in reducing infant mortality rate in the region. The separate effects of private and public health expenditures on this health status indicator were also negative, as expected, and statistically significant, implying that both types of healthcare expenditures are essential for improving the population’s health. However, unlike some earlier studies, Novignon et al. [1] for example, the extent of effect of private health expenditure was found to be higher than that of public health expenditure in the present study. This may be due to the fact that the use of public health funds in these countries is, in general, inefficient due to corruption and inappropriate governance.

Private health care expenditure also significantly decreased the crude death rate in the region, although public health expenditure showed the opposite result. Improper utilisation of public sector funds may be the reason behind this. The present study did not find any significant effect of healthcare expenditure on life expectancy. This may be due to the reason that life expectancy depends on some other important factors such as lifestyle, environment, individual education level, etc., for which we have no available data.

We also found that per capita income growth rate had significant positive effects in increasing life expectancy and reducing infant mortality rates in the region. Improved sanitation facilities also played a significant positive role in increasing life expectancy and decreasing crude death rate and infant mortality rate.

Based on these research findings, the following policy implications may be drawn. Firstly, increases in health expenditure is to be supported in order to improve the health status of the population in the region. The amount of spending is to be more or less close to that of the developed countries. Secondly, proper governance and handling must be upheld for appropriate and efficient use of public sector health funds, and accountability and transparency must be ensured in this regard. Thirdly, efforts should be made and adequate policies must be adopted and executed to increase the income level of the population and to enable them to spend more on health goods and services. Finally, further improved sanitation facilities are to be supported by the government and private initiatives.

The present study faced some limitations, especially when attempt was made to include a large dataset considering a long period of time with many countries, and involving many different variables. For example, the time period of the dataset had to be reduced from 55 years to 20 years, and, the number of countries used from the dataset had to be decreased to 15 from 17 countries. Also, there was no available data on some important variables such as diet, lifestyle, education level and environment, which could have been incorporated as explanatory variables in the models. Future research should address these limitations, though they in no way invalidate the findings of this study.
